# Mining drives extensive deforestation in the Brazilian Amazon

**DOI:** 10.1038/s41467-017-00557-w

**Published:** 2017-10-18

**Authors:** Laura J. Sonter, Diego Herrera, Damian J. Barrett, Gillian L. Galford, Chris J. Moran, Britaldo S. Soares-Filho

**Affiliations:** 1 Gund Institute for Environment, University of Vermont, 617 Main Street, Burlington, VT 05405 USA; 2 Rubenstein School of Environment and Natural Resources, University of Vermont, Burlington, VT 05405 USA; 3 School of Earth and Environmental Sciences, The University of Queensland, Brisbane, Qld 4072 Australia; 4 Centre for Biodiversity & Conservation Science, The University of Queensland, Brisbane, Qld 4072 Australia; 5 Environmental Defense Fund, Washington, DC 20009 USA; 6 Energy Flagship, Commonwealth Scientific and Industrial Research Organisation (CSIRO), Canberra, ACT 2601 Australia; 7 Curtin University, Perth, WA 6102 Australia; 8 Centro de Sensoriamento Remoto, Universidade Federal de Minas Gerais, Belo Horizonte, MG 31270-901 Brazil

## Abstract

Mining poses significant and potentially underestimated risks to tropical forests worldwide. In Brazil’s Amazon, mining drives deforestation far beyond operational lease boundaries, yet the full extent of these impacts is unknown and thus neglected in environmental licensing. Here we quantify mining-induced deforestation and investigate the aspects of mining operations, which most likely contribute. We find mining significantly increased Amazon forest loss up to 70 km beyond mining lease boundaries, causing 11,670 km^2^ of deforestation between 2005 and 2015. This extent represents 9% of all Amazon forest loss during this time and 12 times more deforestation than occurred within mining leases alone. Pathways leading to such impacts include mining infrastructure establishment, urban expansion to support a growing workforce, and development of mineral commodity supply chains. Mining-induced deforestation is not unique to Brazil; to mitigate adverse impacts of mining and conserve tropical forests globally, environmental assessments and licensing must considered both on- and off-lease sources of deforestation.

## Abstract (web summary)

Industrial mining contributes to deforestation in the Amazon, and the extent of effect could occur beyond areas of land explicitly permitted for mining. Here, Sonter et al. show that deforestation in 70-km buffer zones around mines has led to an estimated 9% of Brazilian Amazon deforestation since 2005.

## Introduction

Reducing tropical deforestation to conserve biodiversity and regulate climate is a globally significant goal^1–3^, yet deforestation rates remain high^4^. Even in Brazil, where policy interventions and economic conditions have reduced annual deforestation rates by 80%^5^, >127,000 km^2^ of forests have been cleared since 2005^6^. At present, priority is given to management of spatially extensive drivers of deforestation (e.g., urban population growth and crop production^7, 8^). Demand for minerals also poses significant risks^9, 10^, particularly where mineral resources and biodiverse old-growth forests co-exist^11^ in developing countries that seek revenue from mining but lack regulatory oversight and enforcement capability^12^.

Mining causes deforestation both within and beyond lease boundaries. Within leases, forests are cleared for mineral extraction, processing, and infrastructure development^13, 14^. However, off-lease impacts are potentially more extensive and their pathways more complex^9, 15–18^. Deforestation may extend substantial distances ( > 10 km) beyond lease boundaries^9, 17, 18^, due to the combined effects of land use displacement^19^, urban expansion^20^, development of commodity supply chains^21^, and concerns over mine waste discharge^22^ and spills^23^. It is essential to understand and mitigate mining-induced deforestation if tropical forests are to be conserved^18^, yet the full extent of these impacts (on- and off-lease) have not been quantified heretofore.

**Fig. 1:**
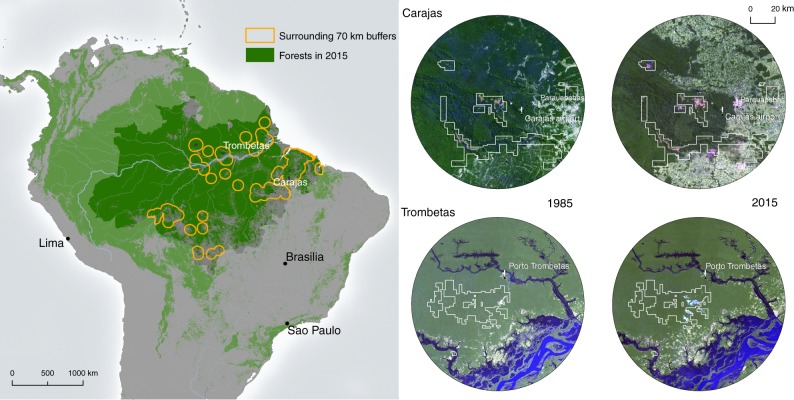
Mining in Brazil’s Amazon forest. *Left*: distribution of mining leases and forests in 2015^4^. *Right*: case study mining operations. Mining leases are shown as white polygons overlying Landsat TM imagery for 1985 and 2015. Landsat pixel-based cloud-free mosaic, bands 3, 2, 1. *Green areas* indicate forests, *bright white areas* indicate non-forested ground, and *blue areas* are water

We focus on Brazil’s Amazon forest (Fig. 1), the world’s largest remaining tropical forest^4^ and a region undergoing increased mining activity^16, 23^. Throughout Brazil, mining leases, concessions, and exploration permits cover 1.65 million km^2^ of land, of which 60% is located in the Amazon forest^24^. Mineral production contributed 4% to Brazil’s gross domestic product in 2011^25^ and this value is projected to increase fourfold by 2030^26^. Approval of new mines and expansion of existing projects require environmental licenses. However, current impact assessments do not systematically consider off-lease, indirect or cumulative sources of deforestation^16^, and proposed legislative changes will expedite approval of strategic projects (654/2,015)^27^, remove the power of environmental agencies to suspend operations based on environmental impacts (PEC-65)^27–30^, and enable extraction within protected and indigenous areas (PL3682/2012; PL1610/1996)^16, 31^.

To quantify mining-induced deforestation, we use satellite data and propensity score matching, a method for identifying causal affects^32–36^. Deforestation (2005–15)^6^ is assessed within ‘treatment’ areas: large operational mining leases (*n* = 50; Fig. 1 and Table 1)^24^ and their surrounding buffers (concentric zones 10 km wide up to 100 km from leases). A control for each treatment is selected from areas farther than 100 km from leases (but within Brazil’s Amazon forest) accounting for spatial variables known to explain the distribution of mining leases^13^ and correlate with deforestation^37^. The difference in forest loss between treatments and these controls is thus the deforestation attributable to mining. Our primary findings are supplemented with two exploratory analyses. First, pathways of mining-induced deforestation are investigated by comparing a range of socio-economic conditions between municipalities that contain mines with those that do not. Second, mining-induced deforestation at the local scale is explored utilizing two case study mines (Carajás and Trombetas). Our results reveal extensive deforestation, significant up to 70 km from lease boundaries, caused by large scale industrial mining operations in Brazil’s Amazon forest.

**Table 1:** Forest cover and deforestation^6^ within mining leases and their surrounding 0–70 km buffers

State	Major commodity	Year	Area (km^2^)	Forest cover 2005 (km^2^)		Deforestation 2005–2015 (km^2^)		Deforestation 2005–2015 (%)	
				ML	0–70 km	ML	0–70 km	ML	0–70 km
AM	Iron ore	1960	16	16	13,430	0	52	0.0	0.4
RO	Tin	1961	150	137	12,398	9	1,826	6.6	14.7
PA	Bauxite	1969	949	879	15,983	36	777	4.1	4.9
PA	Copper	1969	1,400	833	11,527	55	1,838	6.6	15.9
PA	Iron ore	1969	162	80	1,706	32	560	40.0	32.8
PA	Clay	1970	28	0	2,704	0	835		30.9
RO	Tin	1970	96	28	5,681	18	365	64.3	6.4
AP	Bauxite/Kaolin	1971	248	116	12,987	25	474	21.6	3.6
PA	Bauxite	1971	798	465	7,714	56	1,172	12.0	15.2
RO	Tin	1971	950	688	9,544	62	2,524	9.0	26.4
PA	Aluminium	1972	58	4	1,773	2	226	50.0	12.7
MT	Diamond	1972	23	0	2,975	0	591		19.9
PA	Bauxite/Limestone	1973	142	0	1,249	0	222		17.8
PA	Clay	1974	34	0	543	0	75		13.8
AM	Sylvite	1975	408	219	10,724	25	702	11.4	6.5
AM	Limestone	1975	35	35	13,644	0	43	0.0	0.3
PA	Tin	1975	46	14	3,648	7	84	50.0	2.3
PA	Tin	1976	255	124	12,120	61	2,472	49.2	20.4
PA	Bauxite	1977	1,233	833	11,775	133	1,880	16.0	16.0
MT	Dolomite	1977	52	12	2,496	6	640	50.0	25.6
AP	Gold	1978	24	4	7,228	3	37	75.0	0.5
AP	Gold	1978	148	146	13,988	2	16	1.4	0.1
AP	Clay	1978	11	0	2,612	0	55		2.1
AM	Tin	1978	974	879	18,928	29	46	3.3	0.2
MA	Gold	1978	82	24	1,754	5	439	20.8	25.0
PA	Tin	1979	154	83	4,441	40	128	48.2	2.9
MT	Gold	1979	416	106	4,604	33	778	31.1	16.9
AP	Iron ore	1980	100	71	3,285	8	67	11.3	2.0
PA	Limestone	1980	45	30	10,913	7	1,070	23.3	9.8
PA	Bauxite	1980	1,047	379	3,077	153	906	40.4	29.4
RO	Gold	1980	72	41	6,334	9	707	22.0	11.2
MT	Gold	1980	42	23	9,790	17	1,916	73.9	19.6
PA	Gold	1981	78	76	13,500	2	62	2.6	0.5
PA	Gold	1982	22	3	1,748	3	703	100.0	40.2
PA	Silica	1982	63	2	5,524	2	1,617	100.0	29.3
TO	Limestone	1982	13	12	918	3	365	25.0	39.8
MT	Gold	1982	71	4	848	1	215	25.0	25.4
RO	Tin	1987	326	83	4,601	53	1,840	63.9	40.0
AM	Kaolin	1988	260	112	13,716	27	1,122	24.1	8.2
PA	Aluminium	1991	230	129	6,797	10	378	7.8	5.6
MT	Diamond	1991	88	33	11,053	16	1,105	48.5	10.0
AP	Iron ore	1992	210	159	11,437	18	276	11.3	2.4
PA	Kaolin	1993	18	6	1,641	2	476	33.3	29.0
PA	Silica	1995	12	2	2,430	1	652	50.0	26.8
PA	Nickel	1996	148	9	3,856	3	131	33.3	3.4
PA	Silica	2000	22	1	1,353	1	636	100.0	47.0
RO	Tin/Granite	2002	33	9	4,605	8	665	88.9	14.4
MA	Granite	2003	12	7	2,900	0	806	0.0	27.8
RO	Clay	2005	16	0	13,892	0	1,823		13.1
RO	Manganese	2005	30	4	9,987	0	993	0.0	9.9
		Column averages:	237	138	7,048	20	748	33	16
		Column totals:	11,850	6,920	352,381	983	37,388		

Mining leases (ML, n=50) and data on major mined commodities and year in which the operation first received a mining license were obtained from DNPM^24^

## Results

### Extent of mining-induced deforestation

**Fig. 2:**
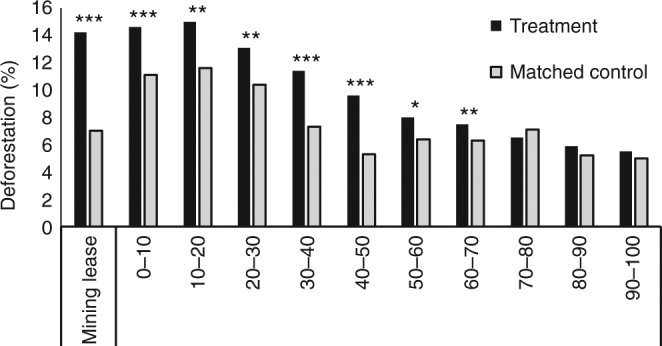
Deforestation rates within treatments and matched controls. Differences between treatments and their matched controls is the deforestation attributeable to mining. *Stars* denote significant differences (*t*-test; ***P < 0.001, **P < 0.01, and *P < 0.05; see Table 2)

Mining caused deforestation within leases. These areas contained 6,880 km^2^ of forests in 2005, of which 14% (983 km^2^) were cleared by 2015 (Table 1). Mining also caused deforestation off-lease. Deforestation within buffers was significantly greater than expected up to 70 km from leases (Fig. 2 and Table 2; *t*-test; *P* < 0.05). Off-lease, forests within the 0–10 km and 40–50 km buffers experienced the highest rates of mining-induced deforestation (4.4%) (Table 2). The total area impacted by mining (lease areas, 0–70 km buffers) contained 337,690 km^2^ of forests in 2005 and underwent 37,830 km^2^ of clearing by 2015 (Table 3). Of this total deforestation, 11,670 km^2^ (31%) was induced by mining (Table 3), as indicated by differences in deforestation rates between treatment areas and their matched controls.

**Table 2:** Deforestation rates within treatments and their matched controls

Treatment	Deforestation rates (2005–2015)			Bias adjusted estimator	Matched controls (n)	Placebo test (t stat)
	Treatment	Matched control	Difference			
Mining lease	0.142	0.070	0.072	0.078***	329	−1.36
0–10 km	0.146	0.111	0.036	0.044***	70	−0.22
10–20 km	0.150	0.116	0.034	0.041**	91	0.82
20–30 km	0.131	0.104	0.027	0.026**	107	1.09
30–40 km	0.114	0.073	0.041	0.042***	90	0.67
40–50 km	0.096	0.053	0.043	0.044***	93	-0.19
50–60 km	0.080	0.064	0.016	0.020*	100	1.33
60–70 km	0.075	0.063	0.012	0.021**	100	0.72
70–80 km	0.065	0.071	−0.006	0.002	107	0.57
80–90 km	0.059	0.052	0.007	0.008	105	−0.18
90–100 km	0.055	0.050	0.005	0.005	125	0.00

‘Difference’ indicates mining-induced deforestation, using the propensity score matching estimator; it represents the difference in deforestation between treatments and their matched controls. The bias adjusted estimator is the mining-induced deforestation when controlling for any remaining post-matching bias (see Methods). ‘Matched controls (*n*)’ are the number of unique control observations used in ‘matching with replacement’. Placebo tests compare matched controls and a placebo set of controls (see Methods); all treatments pass Placebo tests (*P* > 0.05)

*Stars* denote significant differences: ***P < 0.001, **P < 0.01, and *P < 0.05

**Table 3:** Summary statistics for mining leases, surrounding buffers, unmatched controls farther than > 100 km from mining leases, and the total impact area

	Mining leases	Surrounding buffers (km)										> 100 km	Impact area
		0–10	10–20	20–30	30–40	40–50	50–60	60–70	70–80	80–90	90–100		
Land area	11,848	59,069	904,59	150,317	190,610	257,429	304,332	378,685	430,882	506,611	556,179	1,709,020	1,442,749
Forests 2005	6,882	26,738	34,727	42,737	48,908	54,152	59,243	64,303	68,108	68,993	66,158	1,289,961	337,690
Deforestation	983	4,044	5,417	5,808	5,817	5,459	5,105	5,192	4,786	4,379	3,971	76,049	37,825
Mining-induced deforestation	983	1,176	1,424	1,111	2,054	2,383	1,185	1,350	0	0	0	0	11,666
*Covariates*													
Protected areas	0.378	0.384	0.417	0.465	0.480	0.531	0.584	0.607	0.614	0.631	0.640	0.554	
Agricultural suitability	0.117	0.189	0.226	0.271	0.275	0.287	0.308	0.316	0.317	0.342	0.335	0.494	
Distance to rivers	3.312	3.023	2.857	2.849	2.844	2.864	2.884	2.908	2.914	2.939	2.956	3.093	
Elevation	2.610	2.316	2.215	2.227	2.190	2.171	2.172	2.160	2.168	2.174	2.241	2.242	
Distance to roads	4.775	4.505	4.781	4.989	5.106	5.226	5.376	5.467	5.548	5.530	5.503	5.997	
Amazonas	0.149	0.149	0.178	0.209	0.232	0.252	0.256	0.257	0.264	0.256	0.249	0.516	
Rondonia	0.186	0.186	0.188	0.171	0.164	0.159	0.154	0.139	0.107	0.086	0.067	0.020	
Tocantins	0.002	0.003	0.002	0.001	0.003	0.003	0.002	0.003	0.003	0.003	0.002	0.000	
Maranhao	0.022	0.022	0.016	0.029	0.036	0.035	0.030	0.027	0.020	0.017	0.017	0.004	
Para	0.531	0.532	0.464	0.433	0.402	0.387	0.381	0.383	0.391	0.428	0.456	0.235	
Amapa	0.065	0.065	0.082	0.078	0.080	0.081	0.086	0.090	0.085	0.073	0.064	0.010	

The total impact area represents mining leases and 0–70 km surrounding buffers. Top four rows show total land area (km^2^), forest extent in 2005 (km^2^), deforestation between 2005 and 2015 (km^2^), and mining induced deforestation (km^2^). *Bottom rows* show mean covariate values

### Pathways leading to mining-induced deforestation

**Fig. 3:**
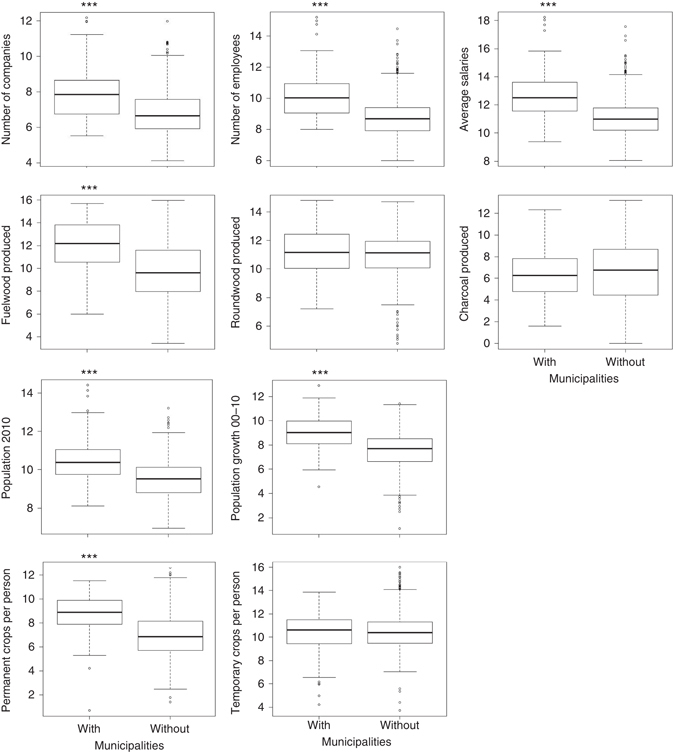
Socio-economic variables of municipalities with mining leases verses those without. All response variables have been log-transformed. *Stars* denote significant differences (Wilcoxon’s rank-sum tests; ***P < 0.001). *Note*: the quantity of fuelwood, roundwood, and charcoal produced (second row) excludes silviculture. All data sets are described in Supplementary Table 3

Socio-economic conditions differed between municipalities that contained mining leases and those that did not (Fig. 3). Municipalities with mines had a greater number of registered companies (*W* = 33,438, *P* < 0.001), employees per company (*W* = 32,594, *P* < 0.001), and salary per employee (*W* = 36,716, *P* < 0.001). Municipalities with mines had a greater population in 2010 (*W* = 35,197, *P* < 0.001) and population growth between 2000 and 2010 (*W* = 34,668, *P* < 0.001). Municipalities with mines also had larger permanent cropped areas (*W* = 26,870, *P* < 0.001) and greater deforestation for fuelwood (*W* = 19,783, *P* < 0.001) but not for roundwood (*W* = 21,581, *P* = 0.311) or charcoal production (*W* = 11,839, *P* = 0.502).

### Evidence from case study mining operations

Carajás, owned by the Brazilian company Vale, is the world’s largest iron ore operation. In 2007, a US $2.48 billion expansion was approved to establish new mining pits and processing infrastructure, clearing forests within leases. An influx of people to support growing operations led to further deforestation, expanding nearby towns and facilities. Mined ore and processed materials are transported via a company-established 890-km-long railway to the deep-water port (Ponta da Madeira) in São Luís, Maranhão. In addition, iron ore mining creates demand for charcoal to produce pig iron, driving expansion of tree plantations and native forest loss^21^.

Trombetas is Brazil’s largest bauxite producer, owned by Mineração Rio do Norte (MRN) and others (Rio Tinto, Vale, South32, CBA, Alcoa, and Norsk Hydro). As with Carajás, factors contributing to deforestation include infrastructure establishment (for mineral processing and transportation) and urban development. For example, bauxite is transported from Trombetas by barge to the Alunorte refinery in the town of Barcarena, where the Porto Trombetas urban area was developed in 1975 to support the growing workforce and catalyze economic development^38^. In addition, off-lease positive environmental changes were evident. For example, the company rehabilitated the Batata Lake and helped establish the Saraca-Taaquera National Forest and Trombetas Biological Reserve to prevent further forest loss and environmental degradation.

## Discussion

Mining caused extensive deforestation in Brazil’s Amazon forest between 2005 and 2015. Deforestation within mining leases was triple the average Amazon clearing rate, caused by the direct consequences of mining. However, mining indirectly caused more extensive deforestation off-lease. By controlling for other spatial determinants of deforestation, we found that these impacts extend 70 km from mining leases—a distance much farther than previously suggested^17^—to affect an area containing 18% of Amazon forests in 2005. In total, mining-induced deforestation has been 12 times greater than that occurring within mining leases alone and caused 9% of all deforestation within Brazil’s Amazon forest since 2005. To capture the full extent of these impacts, the assessment of new mines and expansion of existing projects must consider both on- and off-lease sources of deforestation.

Mitigating off-lease impacts of mining requires understanding the pathways through which this deforestation occurs. In Brazil’s Amazon forest, impact pathways may include mining infrastructure establishment and associated secondary forest clearing (such as that associated with new roads), urban expansion to support a growing workforce and indirect economic activities stimulated by mining. Another pathway is the development of commodity supply chains (e.g., charcoal for iron and steel manufacturing^21^). Specifically, socio-economic statistics^39–42^ and Wilcoxon’s rank-sum tests (Fig. 3) suggest mining operations are associated with increased population growth, greater economic activity, and production of fuelwood and food. Although fuelwood demand is rarely a primary driver of Amazon deforestation, it may represent an intermediate transition between forest and other more profitable land uses.

Mining-induced deforestation was also evident locally, as illustrated in two case study operations from Pará (Fig. 1). Carajás and Trombetas are two of the largest and best-known industrial mining operations in Brazil. Both were established in the 1970s and illustrate long-term ( ± 40 years; Fig. 1) impacts of mining on forests within and beyond leases. We found both operations induced deforestation within 70 km of their leases (Fig. 1), due to urban development and infrastructure establishment for mineral processing and transportation. These operations, as well as other mines analyzed in this study (Table 1), also show that variation exists among them. For some, their impacts extend > 70 km from lease boundaries (such as deforestation due to establishing the 892-km-long railway connecting Carajás to the Ponta da Madeira Port in São Luís) and emerge only after a long period of operation. Case study mines support the findings of this paper, but do not illustrate all possible pathways and consequences of mining-induced deforestation.

The extent and pathways of mining-induced deforestation may also vary among regions^18^. Here we found that mining increased deforestation in Brazil’s Amazon forest, although some companies also invest in conservation (e.g., Alcoa’s efforts at Juruti). However, in the country’s largest iron ore mining region, the Quadrilátero Ferrífero (QF) in Minas Gerais (located outside the Amazon forest), mining companies reduced off-lease deforestation by conserving land adjacent to leases^43, 44^. Mining, deforestation trajectories, and environmental policies differ between these regions, which may explain differing outcomes. Mines in Brazil’s Amazon were often established in previously inaccessible forests near known deforestation frontiers (Fig. 1), which may have drawn people to new economic opportunities and catalyzed further clearing. In comparison, mines in QF compete with, rather than create opportunities for, other land users. Environmental regulation of mining in Minas Gerais is also stricter than those in the Amazon forest. Minas Gerais has additional requirements to avoid, mitigate and monitor impacts and enforcement in this state is effective^45^.

Mining poses significant risks to tropical forests worldwide; yet, rarely is mineral production considered a significant driver of extensive deforestation. Our results reveal important implications for forest conservation in Brazil. Current government policies do not consider the full extent of mining-induced deforestation^16^ and proposed legislative changes further reduce assessment and mitigation requirements^25, 27–31^. Mining-induced deforestation is not unique to Brazil^22^. To conserve tropical forests and mitigate the adverse impacts of mining, assessment, and environmental licensing processes must consider both on- and off-lease sources of deforestation.

## Methods

### Deforestation and spatial determinants

We defined Brazil’s Amazon forest ( ~ 3.1 million km^2^) to include all municipalities intersecting the Amazonian biome^46^. Deforestation in the period 2005–15 (specifically, August 2004–July 2015) was quantified within mining leases and buffer zones using PRODES^6^ time series data resampled to 1 km resolution (nearest neighbor method). Mining leases (*n* = 50; Table 1) were limited to large operations ( > 10 ha), other than quarries, which had been approved prior to 2005^24^. Although some lag time likely exists between lease approval and initial deforestation, this lag is ignored here as it is unlikely longer than 1 year, given that sites often establish site infrastructure prior to final approval. Mining leases were grouped spatially (within 20 km of one another) and by operation to avoid overlap. Nine 10-km-wide concentric buffers were constructed around each lease using ArcGIS version 10.1. We used a 10 km buffer width since impacts of mining have previously been suggested to occur at this distance from lease boundaries^17^.

We obtained data on spatial variables previously found to explain the location of mining operations in Brazil^13^ and correlate with Amazon deforestation^37, 47^. These included the following: protected areas (indigenous lands, strictly protected areas, sustainable use areas, and military areas); agricultural suitability (an indicator of suitability of soil and terrain for mechanized crops^48^); distance to major rivers; distance to major roads; elevation; and state boundaries. Categorical variables (protected areas and agricultural aptitude) are shown in Supplementary Fig. 1. Continuous variables (distance to roads, distance to rivers, and elevation) were categorized to ranges that had been found to significantly impact deforestation, using the Weights of Evidence method (Supplementary Fig. 2). Categorical variables were not auto-correlated ( < 0.3 Pearson’s correlation)^37, 47^ and we assumed no correlation in their errors. Table 3 summarizes land area, forest extent in 2005, deforestation during 2005–15, and spatial variables within mining leases, buffers, and Brazil’s Amazon forest.

### Propensity score matching

Mining leases are biased relative to other non-mining spatial determinants of deforestation. For example, forests within mining leases were less frequently designated as protected areas than forests throughout the rest of Brazil’s Amazon (Table 3). As a result, deforestation observed within surrounding buffers not only reflects the impacts of mining, but also the influence of these other determinants. To control for this observable bias, and quantify mining-induced deforestation, we used propensity score matching. This method has been previously used to quantify the causal effect of protected areas on deforestation within^32^ and beyond^33–35^ their boundaries. Analysis was conducted in Stata 14 (psmatch2)^49^.

For each observation (1 km^2^ grid cell) within a treatment area (mining leases and surrounding buffers), we selected control observations from areas farther than 100 km from leases but within Brazil’s Amazon forest. The ‘matching with replacement’ method was applied, which matches all treatment observations with a control observation, permitting re-use of controls from 1,757,870 potential observations. We used probit models to generate propensity scores^36^ and select control observations that were similar to their treatment’s spatial variables and thus deforestation probability (Supplementary Table 1). To remove any remaining post-matching bias between treatments and controls, we calculated a bias-adjusted estimator by regressing deforestation on the dummy variable for mining leases and all other spatial variables used in the model, using the matched sample (treatment and control observations) (Table 2).

Our method assumes that controlled spatial variables were unaffected by mining and thus represent pre-mining conditions. This was reasonable to assume for exogenous variables (agricultural suitability, elevation, states, and distance to rivers). However, two endogenous variables (distance to roads and protected areas) may affect, or be affected by, mining leases. Forests within buffers were closer to roads and contained fewer protected areas than forests outside buffers (Table 3). If some roads were established to support mining operations, and some protected areas strategically sited away from mineral-rich land, accounting for these effects would increase deforestation within matched controls (Fig. 2). However, we considered the likelihood of these effects to be insignificant. Our road map represents major paved roads and highways^37, 47^, rather than small-scale or mining roads, and decisions to establish new protected areas have only recently considered the location of mining leases^22^. It was not possible to only control for protected areas established prior to mining operations, since 90% of mines were established before the earliest protected areas (< 1970; Table 1). However, removing all observations (treatment and control) from our analysis that fell within protected areas increases estimates of mining-induced deforestation to 20,544 km^2^, representing 16% of all 2005–15 Amazon forest loss (Supplementary Table 2).

We also assume that our matching method controlled for all significant determinants of deforestation. To assess the degree to which our results are sensitive to unobserved heterogeneity we conducted Placebo tests^50^. The placebo group was defined as our matched controls and we matched each of these observations to a unique control observation (matching without replacement). If robust, differences in deforestation between placebos and controls should be insignificant^50^, which was true for all treatments (Table 2). To test model robustness, we repeated all analyses using two alternative matching methods: ‘matching without replacement’ (each treatment observation has a unique control observation) and ‘matching with calipers’ (which sets a threshold propensity score to select control observations). Alternative methods reduced less bias than ‘matching with replacement’ and three buffers (0–30 km) did not pass placebo tests (Supplementary Table 2); thus, we do not report these results in the main paper. However, our main conclusions for both alternative methods—mining-induced deforestation extends beyond lease boundaries and causes more deforestation off-lease than on-lease.

### Pathways leading to mining-induced deforestation

We obtained municipality-scale data on economic activities, population dynamics, and wood and food production (Supplementary Table 3). We hypothesized an indirect association of each of these variables with mining operations. For each variable, we compared municipalities with (*n* = 71) and without mining leases (*n* = 653), using non-parametric Wilcoxon’s rank-sum tests with continuity correction in R version 3.0. Municipalities contained mining operations if they intersected one of the 50 mining leases assessed in this study (Fig. 1 and Table 1).

### Data availability

All data supporting the findings of this study are either publically available online via the referenced source, or can be obtained directly from the corresponding author upon request.

## Electronic supplementary material

Supplementary Information
