# Supplementary material for: Mining drives extensive deforestation in the Brazilian Amazon
Source: Nat Commun. 2017 Oct 18;8:1013. doi: 10.1038/s41467-017-00557-w (PMC5647322; doi:10.1038/s41467-017-00557-w)
Supplement: Supplementary file 1 — Supplementary Information [file 41467_2017_557_MOESM1_ESM.pdf]

Type of file: pdf

Title of file for HTML: Supplementary Information

Description: Supplementary Figures, Supplementary Tables, and Supplementary References

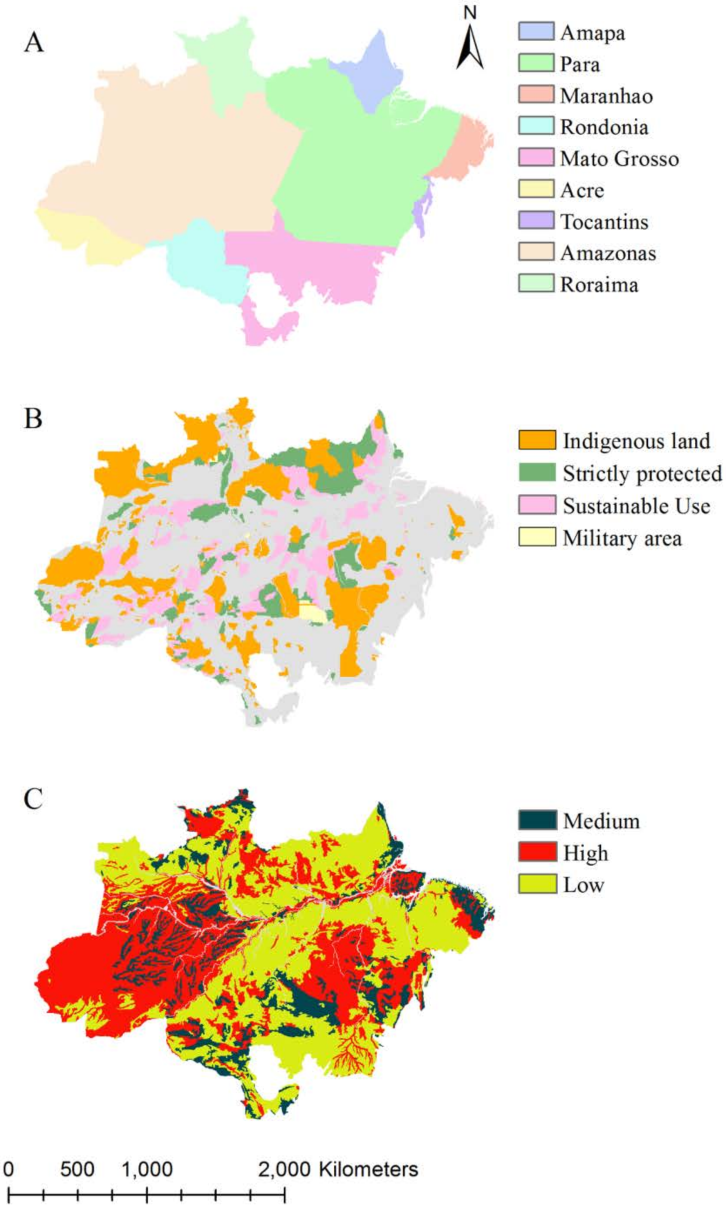

Supplementary Figure 1: Categorical spatial determinants of deforestation, obtained from refs 1 and 2. A: state boundaries; B: protected areas; and C: agricultural suitability, an indicator of suitability of soil and terrain for mechanized crops (produced by ref 3).

Supplementary Figure 2

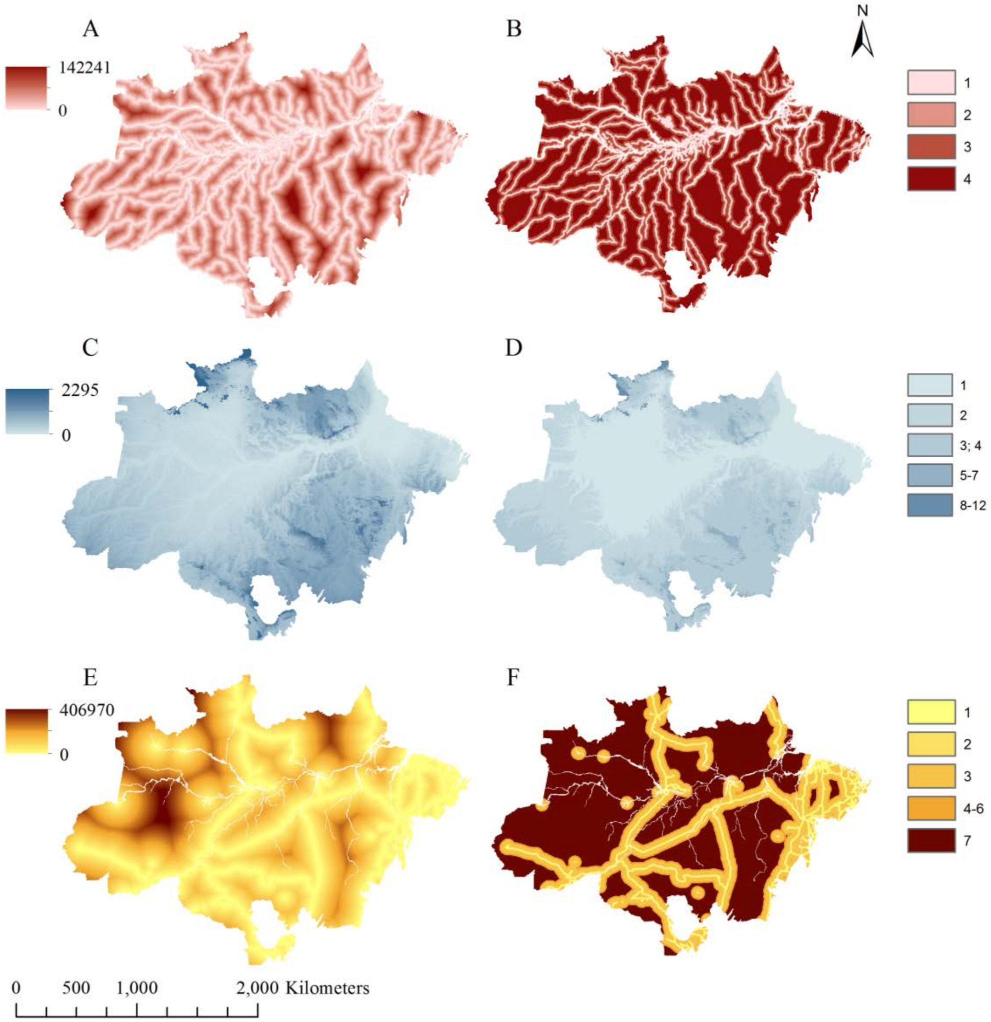

Supplementary Figure 2: Continuous spatial determinants of deforestation and categorical ranges found to significantly impact deforestation, using the Weights of Evidence method as described by ref 2. A: distance to major rivers (m); B: four distance to major rivers categories (1: 0–5,000 m; 2: <60,000 m; 3: <65,000 m; 4: <145,000 m); C: elevation (m); D: nine elevation categories (1: 0–100 m; 2: <200 m; 3: <300 m; 4: <400 m; 5: <500 m; 6: <600 m; 7: <700 m; 8: <800 m; 9: <2,600 m); E: distance to major roads (m); F: seven distance to major roads

categories (1: 0–5,000 m; 2: <25,000 m; 3: <35,000 m; 4:<45,000 m; 5: <60,000 m; 6: <80,000; 7: <410,000 m).

Supplementary Table 1: Covariate balance for 'matching without replacement'. Values represent the bias (%) between treatment and control observations before (U) and after (M) matching for all treatments (mining lease [ML] and surrounding buffers). Controls are >100km from ML. Mean values for treatments and unmatched controls (>100km from ML) are shown in Table 3.

Covariates		ML	Surrounding buffers (km)									
			**0–10**	**10–20**	**20–30**	**30–40**	**40–50**	**50–60**	**60–70**	**70–80**	**80–90**	**90–100**
Protected	U	-35.7	-34.5	-27.4	-17.7	-14.7	-4.6	6.2	10.8	12.3	15.8	17.7
areas	M	3.4	0.7	1.1	1.2	2	2.4	4.8	3.3	4.2	0.6	0.2
Agricultural	U	-89.6	-67.9	-58	-47.2	-46.3	-43.6	-38.7	-37	-36.5	-31.1	-32.8
suitability	M	3.1	2.3	3.5	4.1	3.5	2.8	3.2	1.5	1.5	2.9	0.9
Distance to	U	23.1	-6.7	-22.2	-23.1	-23.4	-22	-20.2	-17.9	-17.2	-14.7	-13
rivers	M	5.9	1	1.5	0.5	0.6	3.3	2.3	0.4	-0.6	0.2	-0.5
Elevation	U	27.6	5.9	-2.3	-1.2	-4.3	-5.9	-6	-7.2	-6.3	-5.8	-0.1
	M	4.8	-3	-3.8	-4.6	-5.9	-5.4	-5.8	-3.9	-2.2	-1.6	-0.5
Distance to	U	-62.8	-75.5	-62	-52.2	-46.3	-40.2	-32.7	-28.1	-24.1	-25	-26.4
roads	M	-5.3	2.9	0.2	4.4	6.2	2.9	7.2	7.6	4.7	1	1
Amazonas	U	-74.6	-84.5	-75.7	-67.2	-61.4	-56.4	-55.4	-55.3	-53.4	-55.3	-57.1
	M	-0.9	-0.1	-0.3	-0.1	-0.1	-0.8	-0.2	0.1	0	0	0
Rondonia	U	45.8	56.8	57.4	53.3	51.3	50.3	49	45.1	36.4	30	23.4
	M	-9.6	-1.1	-3.9	0.7	-4.9	-5.2	-1.9	-0.2	4	0.4	1
Tocantins	U	4.2	-17.8	5.2	-10.1	6.9	6.9	-31.9	-31.2	-20.3	6.9	4.2
	M	-0.4	6.2	-3.3	4.6	-2.9	-4.4	5.1	6.2	6.7	-7.7	-1.4
Maranhao	U	0.2	16.8	12.7	20.5	23.7	22.9	20.9	19.2	15.4	14	13.1
	M	-0.5	-3.7	-5.3	3.9	13.4	11.3	7.8	7.9	-3.1	2.7	-0.5
Para	U	81.2	64.2	49.7	43.1	36.4	33.3	32.1	32.6	34.3	42	47.8
	M	1.3	-2.3	-0.5	-3.2	-2.9	-1.3	-0.7	-0.5	-0.6	-0.8	-0.4
Amapa	U	17.9	29.1	34.8	33.6	34.1	34.5	35.9	37	35.6	32	28.6
	M	15.8	13.6	10.1	5.6	4.6	3.6	4.6	2.8	2.4	1.8	0.6
Average bias post-matching		1.6	-0.68	-0.06	0.22	1.24	0.84	-0.96	-1.11	-0.91	-0.05	0.04

Supplementary Table 2: Deforestation (2005–15) comparisons between treatments (mining leases and surrounding buffers) and matched controls, using alternative matching methods (‘without replacement’, ‘without replacement using callipers’, and ‘with replacement dropping the 40% of treatment observations and 55% of control observations that fell within protected areas’). ‘Matched controls (n)’ represents the number of unique control observations used in ‘matching with replacement’. Matched controls for without replacement methods equal the number of treatment observations (see ‘forest cover in 2005’, Table 3). ‘Difference’ is the difference in deforestation between treatments and matched controls, i.e. the propensity score matching estimator. To control for remaining post-matching bias, we regressed deforestation on the dummy variable for mining leases and all other spatial variables used in the model, using the matched sample (treatment and control observations); this is the bias adjusted estimator. Placebo tests show the t-statistic for comparisons in deforestation rates between controls and a secondary set of matched controls (see Methods).

	**Without replacement**				**Without replacement + callipers (threshold=0.01)**				**With replacement, dropping protected areas**				
	**Deforestation**		**Bias adjusted**	**Placebo (t stat)**	**Deforestation**		**Bias adjusted**	**Placebo (t stat)**	**Matched controls (n)**	**Deforestation**		**Bias adjusted**	**Placebo (t stat)**
**Treatment**	**Cont-rol**	**Diff-erence**			**Cont-rol**	**Diff-erence**				**Cont-rol**	**Diff-erence**		
ML	0.121	0.021	0.023***	0.90	0.121	0.021	0.023***	0.90	211	0.094	0.111	0.109***	-0.61
0–10 km	0.134	0.013	0.011***	4.10**	0.132	0.013	0.011***	3.78**	441	0.159	0.066	0.065***	0.00
10–20 km	0.120	0.030	0.030***	3.89**	0.119	0.031	0.031***	3.68**	469	0.167	0.064	0.066**	0.06
20–30 km	0.114	0.018	0.024***	7.82**	0.113	0.018	0.025***	5.75**	477	0.168	0.047	0.051**	0.59
30–40 km	0.098	0.016	0.017***	0.88	0.098	0.016	0.016***	1.71	515	0.130	0.060	0.062***	0.07
40–50 km	0.087	0.009	0.012***	1.61	0.087	0.009	0.012***	1.52	546	0.098	0.079	0.080***	-0.21
50–60 km	0.078	0.002	0.010***	-0.60	0.078	0.002	0.010***	-0.58	558	0.120	0.047	0.046**	0.07
60–70 km	0.072	0.002	0.011***	-0.58	0.073	0.002	0.011***	1.25	561	0.112	0.052	0.051**	-0.49
70–80 km	0.061	0.004	0.009***	0.09	0.061	0.004	0.009***	-0.2	591	0.126	0.020	0.022	0.49
80–90 km	0.051	0.008	0.008***	-0.80	0.051	0.008	0.008***	-0.3	541	0.113	0.026	0.029	0.73

90–100 km	0.053	0.002	0.002	-0.45	0.053	0.002	0.002	-0.45	538	0.137	0.011	0.012	-0.43

Stars denote significant differences: *** p<0.001, ** p<0.01.

Supplementary Table 3: Socio-economic data used to investigate potential impact pathways of mining induced deforestation. Table shows the years of data collection, and a description of each variable. All variables were collected at the spatial scale of municipalities.

Variable	Reference	Year	Variable	Description
Economic activities	4	Annual data available for years 2006 to 2014. Data from 2012 were used here.	Companies	Total number of registered companies
			Employees	Average number of employees per company
			Salaries	Average salary per employee
Population dynamics	5	Data available for years: 1991, 2000, 2010.	Population	Number of residents in 2010
			Population growth	Change in the number of residents between 2000 and 2010
Wood production	6	Annual data available for 1990 to 2013. Data represent aggregated totals for 2005 to 2014.	Fuelwood	Quantity of fuelwood (m^3) produced from clearing forested land (excludes silvicultural production)
			Roundwood	Quantity of roundwood (m^3) produced from clearing forested land (excludes silvicultural production)
			Charcoal	Quantity of charcoal (t) produced from clearing natural forests
Food production	7	Annual data available for 1990 to 2013. Data represent aggregated totals for 2005 to 2014.	Permanent crops	Area harvested (ha) from permanent crops
			Temporary crops	Area harvested (ha) from temporary crops

Supplementary References

1. Soares-Filho BS, *et al.* Modelling conservation in the Amazon basin. *Nature* **440**, 520-523 (2006).
2. Soares-Filho B, *et al.* Role of Brazilian Amazon protected areas in climate change mitigation. *Proceedings of the National Academy of Sciences* **107**, 10821-10826 (2010).
3. Nepstad DC, Stickler CM, Filho BS, Merry F. Interactions among Amazon land use, forests and climate: prospects for a near-term forest tipping point. *Philosophical Transactions of the Royal Society B: Biological Sciences* **363**, 1737-1746 (2008).
4. IBGE. Cadastro Central de Empresas. Instituto Brasileiro de Geografia e Estatística (2014).
5. IBGE. Censo Demográfico. Instituto Brasileiro de Geografia e Estatística (2014).
6. IBGE. Produção da Extração Vegetal e da Silvicultura. Instituto Brasileiro de Geografia e Estatística (2014).
7. IBGE. Produção Agrícola Municipal. Instituto Brasileiro de Geografia e Estatística (2014).
